# Curing Benign Paroxysmal Positional Vertigo in Patients With Brain Tumor: Case Series and Literature Review

**DOI:** 10.7759/cureus.13873

**Published:** 2021-03-13

**Authors:** Khalid Bashir, Mohammed T Bashir, Amr Elmoheen

**Affiliations:** 1 Emergency Medicine, Qatar University College of Medicine, Doha, QAT; 2 Emergency Medicine, Hamad Medical Corporation, Doha, QAT; 3 Emergency Medicine, Weill Cornell Medical College Qatar, Doha, QAT; 4 School of Medicine and Dentistry, University of Aberdeen, Scotland, GBR

**Keywords:** benign paroxysmal positional vertigo, vestibular disease, dizziness, semi-circular canal, brain tumors

## Abstract

Benign paroxysmal positional vertigo (BPPV) is a common vestibular disorder. It accounts for a third of all vestibular disorders diagnosed in the general population and is usually diagnosed and treated successfully. This article presents two cases of BPPV in a 52-year-old man and a 45-year-old patient, respectively. Both patients presented with recurrent episodes of vertigo associated with certain head movements. Medical history for the first patient included surgery for acoustic neuroma 12 months before developing the vertigo episodes. The second patient underwent a neurosurgical operation for glioblastoma multiforme (GBM) followed by radiotherapy three months before this presentation.

Both patients were diagnosed with right-sided posterior canal BPPV after the Dix-Hallpike test. Their symptoms completely resolved within few minutes of the Epley maneuver. These cases highlight the importance of diagnosing and treating a potentially curable condition that can coexist in some patients with brain tumors.

## Introduction

Benign paroxysmal positional vertigo (BPPV) is a vestibular condition that affects up to nine percent of adults between the ages of 18-35 years [1]. The main characteristics of BPPV are short episodes of vertigo due to head movements as well as nystagmus due to position changes [2].

Most cases of BPPV have idiopathic causes. But in some cases, the BPPV may be caused by vestibular neuritis, head trauma, labyrinthitis, vertebrobasilar ischemia, prolonged bed rest, or as a complication of middle ear surgery. Patients between the ages of 50-70 usually experience idiopathic BPPV, and the condition has a higher prevalence among males than females [3].

Vertigo episodes commonly experienced by patients are due to an imbalance in the inner ear [3, 4-7]. The inner ear is an important component of the vestibular system. Its major functions are: stabilizing visual images during head movements, promoting clear vision, maintaining the stability of posture during head (and other) movements, and providing feedback that enhances proper spatial orientation [6]. The vestibular labyrinth serves as the inner ear mechanism. Crystalline structures known as otoconia lie within the utricle. These crystals are believed to disconnect from the utricle and fall into the semi-circular canals, leading to vertigo symptoms [3, 6, 7].

It is important to note that there are three semi-circular canals within the middle ear, and all are prone to get affected by BPPV. These are the anterior canal, the horizontal, and the posterior canal. All three canals respond to angular acceleration within a plane precisely 90 degrees from each other, thus allowing accurate spatial orientation in all three planes [6].

BPPV presents with vertigo-like symptoms accompanied by nausea and vomiting. Vertigo lasts for up to a minute, while nausea and vomiting may not be present in all cases. Most patients report vertigo-like episodes when lying down, extending their head or neck, sitting up from a supine position, and bending over. Vertigo episodes affect the patient’s balance, but the balance should remain normal between vertigo periods. Also, the horizontal canal involvement may cause the patient to experience an episode with turning over from side to side in a supine position [3, 6, 7].

A major diagnostic symptom of BPPV is nystagmus [4]. It is an eye twitch that occurs during a quick change of the head’s position. The nystagmus is usually assessed with the Dix-Hallpike maneuver [8]. Depending on the canal that is involved, movement of the nystagmus will occur but in specific directions with a particular head movement indicating which canal is involved. Treatment is then targeted at the affected canal with the right canalith repositioning technique [6, 9].

Treatment is mostly done with Epley’s canalith repositioning technique [4, 10, 11]. A secondary treatment known as the Brandt-Daroff exercise is usually taught to patients as a home treatment technique. This is necessary only if symptoms persist after treatment but are not so severe that clinical professionals are needed [3, 12].

## Case presentation

**Figure 1:**
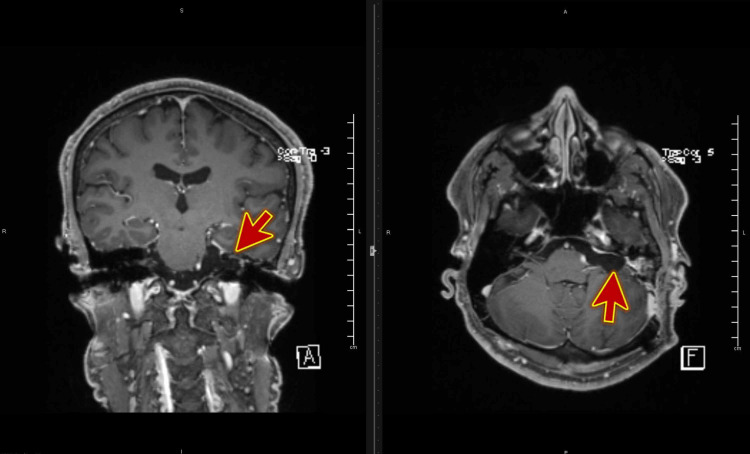
MRI scan of the head with contrast showing postoperative images of left acoustic neuroma.

A 52-year-old man presented to the dizziness clinic with four months history of recurrent episodes of vertigo associated with certain head movements. His significant past medical history includes acoustic neuroma surgery 12 months before developing these vertigo episodes (Figure 1).

During these past four months, he visited several private clinics where he was given a different type of medication (prochlorperazine, Betahistine HCL) which provided temporary relief. In the dizziness clinic, his vital signs and neurological examination were unremarkable, and he was diagnosed with right-sided posterior canal BPPV after the Dix-Hallpike test. His symptoms completely resolved within few minutes of Epley maneuver and remained symptom-free for four months after the treatment.

**Figure 2:**
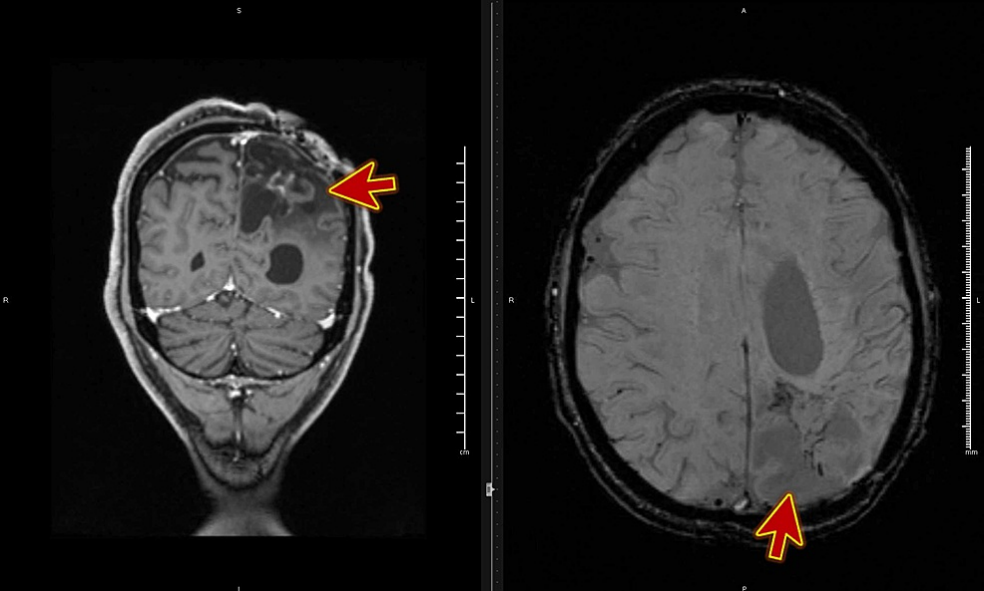
MRI scan of the head with contrast showing postoperative images of glioblastoma multiforme.

A 45-year-old patient was admitted to a medical ward following two weeks of recurrent episodes of vertigo associated with certain head movements up and right side. He underwent a neurosurgical operation for glioblastoma multiforme (GBM) followed by radiotherapy three months before this presentation (Figure 2).

He was chair-bound but was able to move with the helper in a wheelchair. While in the wheelchair, he developed vertigo every time he moved his head up or to the right. He had MRI brain and blood tests during the hospital stay, which didn’t reveal any new abnormality. His brain MRI was similar to the one done three months previously, with no new change. An otolaryngologist assessed him. Diagnosis of right-sided posterior canal BPPV was made after the Dix-Hallpike test and was treated with Epley maneuver with complete resolution of symptoms. He remained vertigo-free three months after the treatment.

## Discussion

This case series is a clear illustration of the Epley maneuver’s effectiveness against BPPV. The patients in this study were middle-aged and treated for underlying comorbidities, thus giving room for optimal Epley maneuver results. Due to no return of symptoms for an extended period (4 and 3 months, respectively), it is assumed that both patients received successful treatments in a couple of maneuver sessions. It is slightly faster compared to results from previous researches. Studies have shown that even a single maneuver is sufficient to complete the treatment of a posterior canal [13].

BPPV diagnosis was verified with nystagmus’ presentation after the Dix-Hallpike maneuver’s performance on the right side. Studies have shown that the direction of the nystagmus may serve as a pointer to the affected canal. Specifically, right-sided Dix-Hallpike nystagmus should be indicated by an upbeat clockwise motion [9]. We did not collect the nystagmus’ specifics present in our patients, but we observed that they were present in the position appropriate for right-sided posterior canal BPPV indication.

Based on this report, we can conclude that posterior canal BPPV may be diagnosed via the Dix-Hallpike maneuver if nystagmus is present. Patients involved may receive one to three sessions of the Epley maneuver, focusing on the diagnosed side [9, 13]. Research suggests the needlessness of postural restriction after treatment but instead harps on implementation of the Brandt-Daroff exercises by the patient, if necessary [9, 12]. BPPV is a common medical problem and can co-exist with other medical conditions including serious pathologies such as brain tumors. BPPV can be effectively treated by bedside maneuvers.

## Conclusions

Patients who present with vertigo symptoms, despite having intracranial pathology should be investigated for BPPV. Based on the patient’s history and the emotions that yield the most intense symptoms, it is essential to employ the Dix-Hallpike maneuver to determine BPPV diagnosis. After the test is administered and nystagmus is indicated, the clinician may confirm the involvement side. Treatment is focused on the side indicated. The Epley maneuver should be employed as treatment.
